# Novel STAT3 Inhibitor LDOC1 Targets Phospho-JAK2 for Degradation by Interacting with LNX1 and Regulates the Aggressiveness of Lung Cancer

**DOI:** 10.3390/cancers11010063

**Published:** 2019-01-09

**Authors:** Chia-Huei Lee, Ji-Rui Yang, Chih-Yu Chen, Ming-Hsien Tsai, Pin-Feng Hung, Shin-Jih Chen, Shang-Lun Chiang, Han Chang, Pinpin Lin

**Affiliations:** 1 National Institute of Cancer Research, National Health Research Institutes, 35 Keyan Road, Zhunan, Miaoli County 35053, Taiwan; jry7921@gmail.com (J.-R.Y.); chihyu0128@gmail.com (C.-Y.C.); hdp91111@nhri.org.tw (P.-F.H.); 070510@nhri.org.tw (S.-J.C.); 2 National Institute of Environmental Health Science, National Health Research Institutes, 35 Keyan Road, Zhunan, Miaoli County 35053, Taiwan; mhtsai@nhri.org.tw; 3 Environment-Omics-Disease Research Center, China Medical University Hospital, Taichung 40402, Taiwan; chimpanzee99999@gmail.com; 4 Department of Health Risk Management, College of Public Health, China Medical University, Taichung 40402, Taiwan; 5 Department of Pathology, China Medical University Hospital, No. 2, Yude Road, North District, Taichung 40447, Taiwan; changhan@mail.cmu.edu.tw

**Keywords:** LDOC1, JAK2, STAT3, lung cancer, ubiquitination, LNX1, lung cancer

## Abstract

Meta-analysis revealed that *Leucine Zipper Down-Regulated In Cancer 1* (*LDOC1*) increased methylation more in people with lung tumors than in those who were healthy and never smoked. Quantitative methylation-specific PCR revealed that cigarette smoke condensate (CSC) exposure drives *LDOC1* promoter hypermethylation and silence in human bronchial cells. Immunohistochemistry studies showed that *LDOC1* downregulation is associated with poor survival of patients with lung cancer. Loss and gain of LDOC1 functions enhanced and attenuated aggressive phenotypes in lung adenocarcinoma A549 and non–small cell lung carcinoma H1299 cell lines, respectively. We found that LDOC1 deficiency led to reinforcing a reciprocal loop of IL-6/JAK2/STAT3, through which LDOC1 mediates the cancer progression. LDOC1 knockdown considerably augmented tumorigenesis and the phosphorylation of JAK2 and STAT3 in vivo. Results from immunoprecipitation and immunofluorescent confocal microscopy indicated that LDOC1 negatively regulates JAK2 activity by forming multiple protein complexes with pJAK2 and E3 ubiquitin-protein ligase LNX1, and in turn, LDOC1 targets pJAK2 to cause ubiquitin-dependent proteasomal degradation. LDOC1 deficiency attenuates the interactions between LNX1 and pJAK2, leading to ineffective ubiquitination of pJAK2, which activates STAT3. Overall, our results elucidated a crucial role of LDOC1 in lung cancer and revealed how LDOC1 acts as a bridge between tobacco exposure and the IL-6/JAK2/STAT3 loop in this human malignancy.

## 1. Introduction

Lung cancer is a leading cause of cancer deaths in men and women. Lung cancer is heterogeneous and is classified into two types, small-cell lung carcinomas and non-small-cell lung carcinomas (NSCLCs), based on tumor histology. NSCLCs account for 85% of lung cancer cases and constitute heterogeneous subtypes of squamous, adenocarcinoma, and large-cell carcinomas [1]. Despite progress, lung cancer therapeutic outcomes remain unsatisfactory. Smoking tobacco is recognized as the most prominent risk factor and accounts for approximately 87% of lung cancer cases [2].

In another study, we identified *LDOC1* as an X-linked tumor suppressor and revealed that it is frequently silenced by promoter hypermethylation in oral squamous cell carcinoma (OSCC) in patients who habitually drink alcohol, chew betel quid, or smoke cigarettes [3]. We also discovered that promoter methylation of *LDOC1* is sensitive to cigarette exposure in human untransformed oral keratinocytes [4]. The *LDOC1* gene encodes a protein of 146 amino acids with a typical leucine-zipper motif in the N-terminal domain and a proline-rich region that shares a marked similarity to an SH3-binding domain [5]. These two domains may confer versatile *LDOC1* cellular functions through interaction with various cellular proteins [6,7]. Although *LDOC1* is ubiquitously expressed in all tissues but downregulated or silenced in many cancer types—including cervical cancer [8], ovarian cancer [9], OSCC [3], papillary thyroid carcinoma [10], and osteosarcoma [11]. In these human cancers, *LDOC1* functions as a tumor suppressor by inhibiting proliferation and metastasis and by inducing apoptosis. However, its oncogenic role has been observed in chronic lymphocytic leukemia, in which a high level of *LDOC1* expression predicts poor overall survival [12]. In addition to modulating tumor biology in several human malignancies, *LDOC1* participates in innate immune response and homeostasis of the intestinal mucosa [2]. Furthermore, *LDOC1* is essential in placentogenesis, acting as a long terminal repeat retrotransposon [13,14,15] and affecting reproductive fitness by regulating placental endocrine function [16]. Using meta-analysis, we revealed that *LDOC1* expression is notably downregulated in non-cancerous and cancerous lung tissue in smokers [4]. However, the effect of *LDOC1* in lung cancers has not been elucidated. Given the close association between cigarette smoke and lung cancers, we proposed that *LDOC1* may play a role in the pathogenesis of lung cancers.

## 2. Results

### 2.1. LDOC1 Was Silenced by Promoter Hypermethylation in a Cigarette Smoke Condensate (CSC)-Exposed BEAS-2B Cell Line and Was Associated with the Clinical Outcome of Patients with Lung Cancer

**Figure 1:**
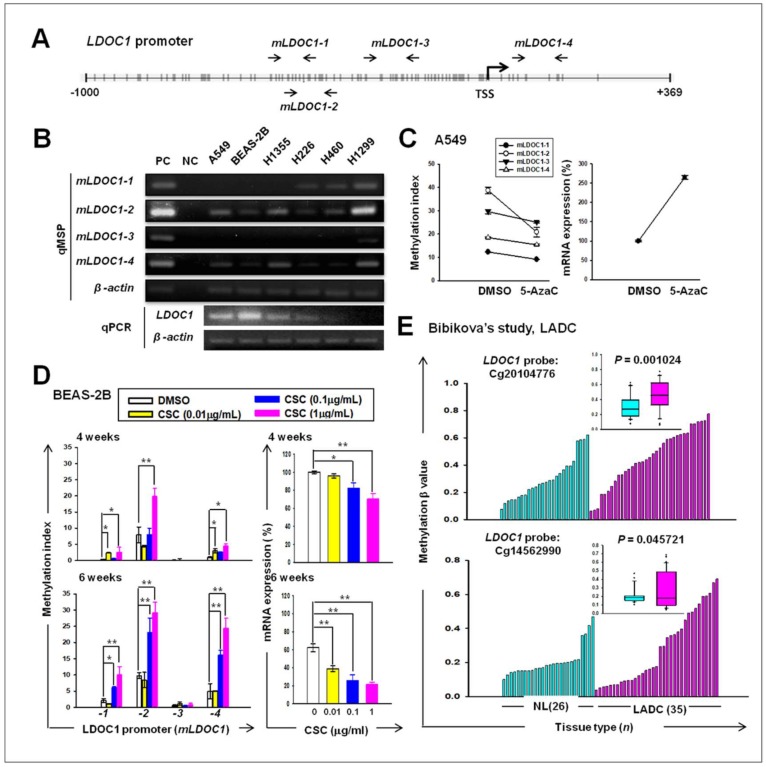
Effect of cigarette smoke on the expression and promoter methylation of *Leucine Zipper Down-Regulated in Cancer 1* (*LDOC1*) in human lung cells. (**A**) Locations of quantitative Methylation-Specific PCR (qMSP) primer pairs in the CpG island proximal to the LDOC1 transcription start site. (**B**–**D**) The promoter DNA methylation (upper in B, left in C and D) and the mRNA expression (bottom in B, right in C and D) of LDOC1 in lung cancer cell lines and BEAS-2B bronchial epithelial cells (**B**) or in the A549 lung adenocarcinoma(LADC) alveolar basal epithelial cell line with or without 5AzaC treatment (**C**) or in BEAS-2B cells after exposure to the indicated concentrations of cigarette smoke condensate (CSC) for 4 or 6 weeks (**D**) were measured using qMSP and qPCR, respectively. Dimethyl sulfoxide (DMSO) treatment was used as control. Universally methylated and unmethylated DNA (Chemicon) were used as positive (PC) and negative controls (NC) in qMSP, respectively. Data are presented as mean ± SD (*n* = 3), analyzed using a Student’s *t*-test. * *p* < 0.05; ** *p* < 0.01. (**E**) The DNA methylation profiles of LDOC1 in LADC [17] were obtained using TCGA.

The genomic locations of the four primer pairs used in qMSP for *LDOC1* are shown in Figure 1A. *LDOC1* was downregulated in all five lung cancer cell lines that were examined relative to the high *LDOC1* level in BEAS-2B cells (Figure 1B). Results from qMSP indicated that the CpG-rich regions of *LDOC1* promoter, *mLDOC1-2* and *mLDOC1-4*, were apparently methylated in lung cancer cell lines A549, H1355, and H1299, with additional methylated *mLDOC1-1* presented in H1299, which show *LDOC1* as completely silenced. Methylation of these three CpG-rich regions was undetectable in BEAS-2B cells (Figure 1B). These data suggested a reverse relationship between promoter methylation and gene expression of *LDOC1* in all human lung cell lines tested. Treatment with 5-AzC, an inhibitor of DNA methyltransferases (DNMTs), transcriptionally reactivated *LDOC1* following promoter DNA demethylation (Figure 1C). The methylation of *mLDOC1-1*, *mLDOC1-2*, and *mLDOC1-4* increased progressively and was accompanied by the progressive downregulation of *LDOC1* mRNA expression in the BEAS-2B cells following exposure to CSC for 4 and 6 weeks in a dose- and time-dependent manner (Figure 1D). DNA methylation array data for 35 lung adenocarcinoma (LADC) and 26 healthy lungs from the Cancer Genome Atlas (TCGA) indicated that the methylation index of two probes mapped to *LDOC1* CpG islands were significantly increased in LADC samples compared with healthy lung tissue (*p* = 0.001024 and 0.045721, respectively; Figure 1E). Collectively, these data indicated that *LDOC1* is a susceptible epigenetic target when human respiratory tracts are exposed to cigarette smoke and suggested that *LDOC1* plays a possible role in the malignant progression of lung cancer.

**Figure 2:**
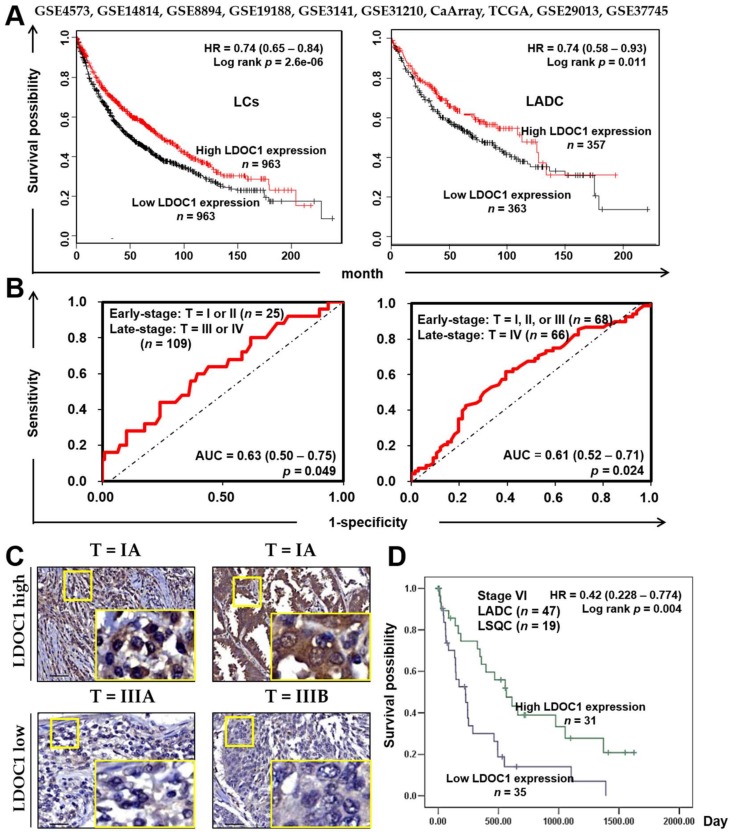
Prognosis analysis according to mRNA and protein expression of *LDOC1* in lung cancer specimens. (**A**) Online Kaplan–Meier survival analysis (http://kmplot.com/analysis) of ten integrated published microarray datasets from lung cancer (LC, left) or LADC (right) specimens according to high or low LDOC1 mRNA expression levels (LC, *p* = 2.6 × 10^−6^; LADC, *p* = 0.011; log-rank test). (**B**) Receiver operating characteristic (ROC) curves for LDOC1 protein expression and T stage of lung cancer specimens in a TMA. Specimens were classified into early-stage (T = I or II (left); T = I, II, or III (right)) and late-stage (T = III or IV (left); T = IV (right)). The areas under the Receiver Operating Characteristic (ROC) curve (AUC) with 95% confidence interval (CI) are indicated. (Left, *p* = 0.049; right, *p* = 0.024; *t*-test). (**C**) Representative immunohistochemistry images indicate high LDOC1 expression in stage IA but low LDOC1 expression in stage IIIA and IIIB lung cancer specimens. Small and large yellow frames indicate the original and magnified areas, respectively. Scale Bar, 50 μm. (**D**) Kaplan–Meier survival analysis of patients with lung cancer with stage IV specimens (*n* = 66, including LADC (*n* = 47) and lung squamous cell carcinoma (*n* = 19)) in a TMA according to high or low LDOC1 expression levels determined by IHC (*p* = 0.004; log-rank test).

We investigated the association between LDOC1 expression and prognosis in two patient cohorts. First, a cohort of patients (*n* = 1926) with lung cancers encompassing all subtypes from an integrated database of ten published transcriptomic datasets [18] was subjected to an online Kaplan–Meier overall survival (OS) analysis (http://kmplot.com/analysis), which revealed a strong association (*p* = 2.6 × 10^−6^, log-rank test, *n* = 1926; Figure 2A, left) between low LDOC1 mRNA levels (≥median versus <median) and poor OS. A significant association (*p* = 0.011, log-rank test, *n* = 866; Figure 2A, right) between low LDOC1 mRNA levels and poor OS was also identified among patients with LADC in this cohort. Next, we performed immunohistochemistry (IHC) with a tissue microarray (TMA) prepared from lung tumor specimens of another patient cohort (*n* = 129). Receiver operating characteristic (ROC) curves for tumor clinicopathological features, including T stage, N stage, and tumor grade, were constructed to determine a discriminative cutoff score for the LDOC1 IHC. LDOC1 majorly localized at the cytoplasm of epithelial lung cancer cells and was not detected in the cellular stroma. Weak or silenced LDOC1 expression was observed in 60.4% of the lung cancer tissues, whereas 39.5% of the lung cancer tissues exhibited LDOC1 immunostaining. ROC analysis revealed that LDOC1 had a high discrimination power (0.63 or 0.61) for T stage features [early (T1 + T2) vs. late (T3 + T4), *p* = 0.049 (Figure 2B, left) or early (T1 + T2 + T3) vs. late (T4), *p* = 0.024 (Figure 2B, right), respectively. The representative images of IHC, which indicated that LDOC1 protein expression was negatively correlated with clinical stages of lung cancer, are shown in Figure 2C. Kaplan–Meier analysis demonstrated that patients with stage IV lung cancer expressing low LDOC1 protein levels had a significantly shorter (*p* = 0.004, log-rank test) OS (mean 351 ± 79 days) than patients expressing high LDOC1 protein levels (mean 742 ± 115 days) (Figure 2D). These results indicated that downregulation of LDOC1 in lung cancer was associated with a poor prognosis, especially for shorter OS, suggesting that LDOC1 is a potential biomarker for the clinical outcome of patients with lung cancer.

### 2.2. LDOC1 Knockdown Mediates Malignancy Progression of Lung Cancer In Vitro

**Figure 3:**
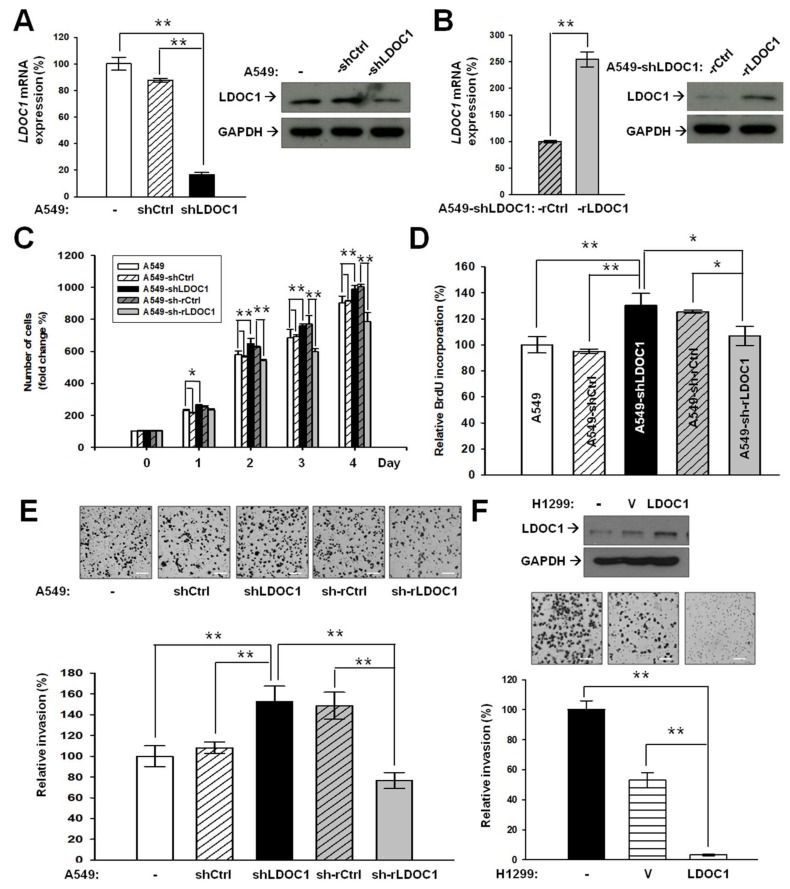
*LDOC1* mediates the in vitro malignancy progression in lung cancer cell lines. (**A**) Expression of *LDOC1*mRNA and protein were stably suppressed in the A549-derived cell lines. The A549 cell line was transducing lentivirus carrying either scramble control vector (A549-shCtrl) or siRNA targeting *LDOC1* (A549-shLDOC1). (**B**) Restoration of *LDOC1* expression in A549-shLDOC1-derived cells. A549-shLDOC1 was only transducing GFP-tagged lentivirus vectors (A549-sh-rCtrl) or those fused with an *LDOC1* open reading frame (A549-sh-rLDOC1). Expression of *LDOC1*mRNA (left) and protein (right) was measured using qPCR and Western blotting analysis, respectively. (**C**–**F**) Cell proliferation (**C**, by MTT), cell cycle progression (**D**, by BrdU incorporation assay), and cell invasion (**E**,**F** by Matrigel coated trans-well invasiveness assay) were assessed in the parental A549 cells and the generated A549-derived cell lines (**C**–**E**) or H1299 ectopic-expressing *LDOC1* (**F**). H1299 cells were transduced with lentivirus carrying either open reading frame (ORF) of *LDOC1* or empty vector (V, controls). Expression of LDOC1 and GAPDH proteins were measured using Western blotting analysis (**F**, upper panels). The number of invading cells was the average of four independent experiments; data were analyzed using a Student’s *t*-test., where * *p* < 0.05 and ** *p* < 0.01. Representative photographs of the invading cells are presented. Scale Bar, 1 mm.

A549-derived cell lines, A549-shLDOC1 and A549-sh-rLDOC1 (with *LDOC1* knockdown expression and restored expression, respectively), as well as their corresponding controls, A549-shCtrl and A549-sh-rCtrl, were generated. The expression of *LDOC1* mRNA and protein in these A549-derived cell lines were confirmed (Figure 3A,B). Our results indicated that *LDOC1* knockdown considerably increased the proliferative abilities of A549-shLDOC1 cells compared with the parental A549 and control A549-shCtrl cell lines, whereas enforced *LDOC1* expression considerably suppressed the proliferation of A549-sh-rLDOC1 cells compared with A549-sh-rCtrl cells (Figure 3C). Results from a BrdU incorporation assay confirmed that the cell cycle progression of A549 can be modulated by *LDOC1* expression levels (Figure 3D). *LDOC1* knockdown led to considerably increased invasiveness of A549-shLDOC1, whereas little enhancement effect was observed in A549-shCtrl (Figure 3E) compared with A549. Compared with A549-shLDOC1 cells, restored expression of *LDOC1* markedly suppressed the invasion of A549-sh-rLDOC1 cells, where the inhibitory effect was as minor as that observed in A549-sh-rCtrl cells (Figure 3E). Overexpression of *LDOC1* drastically suppressed the invasion of H1299 (Figure 3F), highlighting the inhibitory effect of *LDOC1* in trans-well invasiveness.

### 2.3. LDOC1 Knockdown Activated a Reciprocal Loop of IL-6/JAK2/STAT3, through Which LDOC1 Mediated the Aggressiveness of Lung Cancer Cells

**Figure 4:**
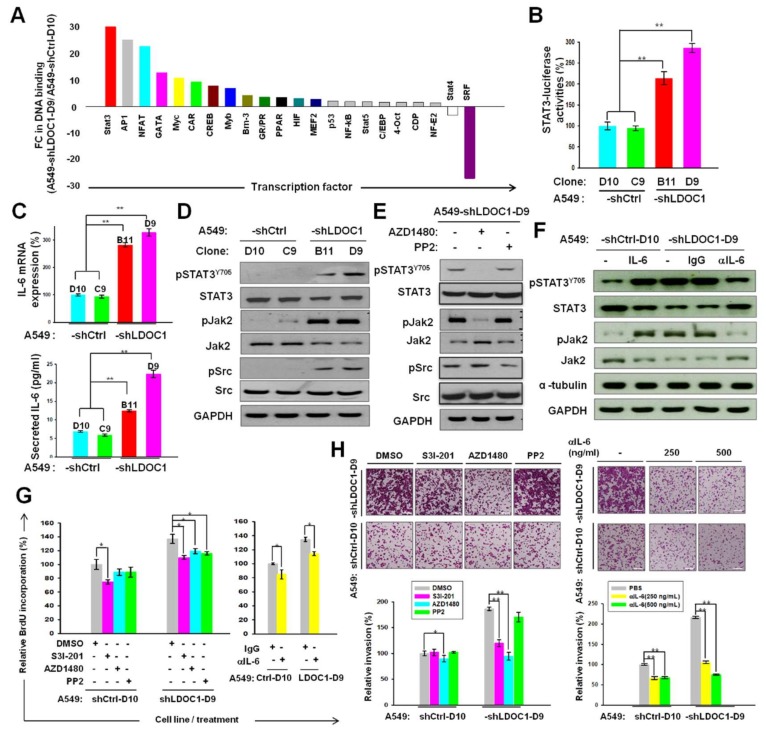
Silence of *LDOC1* induced a reciprocal activation loop of IL-6/JAK2/STAT3, through which *LDOC1* mediated cell cycle progression and invasiveness of lung cancer cells. (**A**) *LDOC1* modulates DNA binding activities of several transcription factors (TFs) in A549 cells. TF activation profiling assays were performed with nuclear extracts of A549-shLDOC1-D9 and A549-shCtrl-D10 cells. The values derived from TFII were set as 1 and used for normalization. The fold change (FC) of TF DNA binding activities in A549-shLDOC1-D9 versus A549-shCtrl-D10 was calculated using the average of two independent experiments. (**B**,**C**) *LDOC1* expression regulates the transcriptional activities of STAT3 (**B**, luciferase reporter assay) and IL-6 mRNA expression (**C**, upper, qPCR) and secretion (**C**, bottom, enzyme-linked immunosorbent assay). Data are presented as mean ± SD (*n* ≥ 3), analyzed using a Student’s *t*-test, where ** *p* < 0.01 versus controls. (**D**) *LDOC1* knockdown induces phosphorylation of STAT3^Y705^, JAK2, and Src in A549-shLDOC1-B11 and -D9 clones. (**E**) Tyrosine kinase activity of JAK2, not Src, is required for phosphorylation of STAT3^Y705^ in A549-shLDOC1-D9 cells. (**F**) Autocrine IL-6 signaling is involved in phosphorylation of JAK2 and STAT3^Y705^ in A549-shLDOC1-D9 cells. Cellular protein lysate was harvested after treatment with inhibitors of JAK2 (AZD1480, 0.5 μM), Src (PP2, 5 μM) (**E**), recombinant human IL-6 (100 ng/mL), or IL-6 neutralizing antibodies (250 ng/mL) (**F**) for 4 h, followed by Western blotting analysis of pSTAT3^Y705^, STAT3, pJAK2, JAK2, GAPDH, and α-tubulin. Cells treated with DMSO (0.1%, **E**) or IgG (250 ng/mL, **F**) were used as controls. Cell cycle progression (BrdU incorporation) (**G**) and trans-well invasiveness (**H**) ofA549-shCtrl-C9 and A549-shLDOC1-D9 were assessed in the presence or absence ofS3I-201 (10 μM), AZD1480 (0.5 μM), or PP2 (5 μM) or indicated concentrations of IL6-neutralizing antibodies. The invasiveness was determined and quantified as described in Figure 3. Representative photographs (upper) and quantification (bottom) of invading cells are shown. Scale Bar, 1 mm. Data are presented as mean ± SD, obtained from four independent experiments, where * *p* < 0.05 and ** *p* < 0.01.

**Figure 5:**
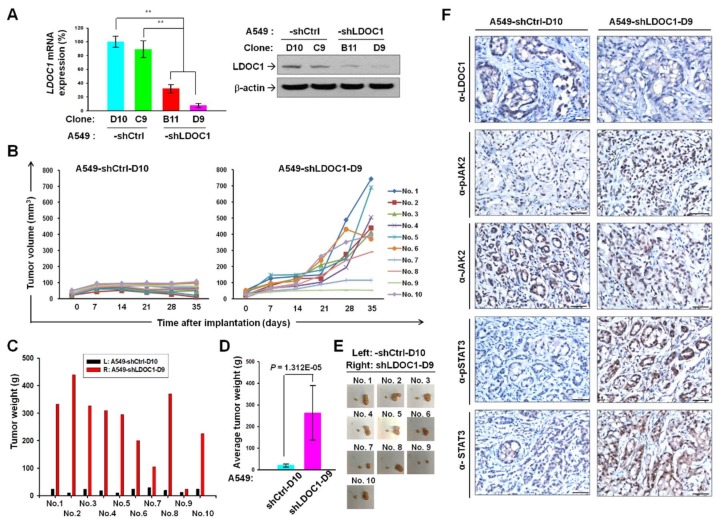
Reduction in *LDOC1* expression promoted tumor growth in subcutaneous xenograft models of lung cancer. (**A**) Expression of *LDOC1* mRNA (left) and protein (right) in A549-derived clones stably suppressed either *LDOC1* expression (A549-shLDOC1-B11 and -D9) or knockdown controls (A549-shCtrl-D10 and-C9). ** *p* < 0.01. (**B**–**E**) A tumor xenograft experiment was performed by subcutaneously injecting A549-shLDOC1-D9 and A549-shCtrl-D10 cells into the right and left back of Balb/c nu/nu mice, respectively. (**B**) The volumes of xenograft tumors in the mice were measured as described in Materials and Methods. The individual (**C**) and the average (**D**) tumor weight (data indicated mean tumor weight ± SD, *n* = 10) and the images (**E**) of harvested tumors derived from A549-shLDOC1-D9 and A549-shCtrl-D10 cells on each mouse were compared on day 35. (**F**) Representative immunostaining of LDOC1, pJAK2, JAK2, pSTAT3^Y705^, and STAT3 in xenograft tumors derived from A549-shLDOC1-D9 and A549-shCtrl-D10 cells. Scale Bar, 50 μm.

Given the potential interaction with basic leucine-zipper transcription factors (TFs), we speculated that *LDOC1* exerts a tumor suppression function through transcriptional regulation. We conducted a TF DNA-binding profiling assay with nuclear proteins isolated from A549-shCtrl-D10 and A549-shLDOC1-D9 to identify LDOC1-regulated TFs. Of the total 22 LDOC1-mediated TFs identified (2 and 20 decreased (fold change [FC] < 0.5) and increased (FC > 1.5) in DNA-binding ability in A549-shLDOC1-D9 cells, respectively), STAT3 was one of the most activated (FC = 30.1) TFs by *LDOC1* knockdown (Figure 4A). The luciferase reporter assay confirmed the induction effect of *LDOC1* knockdown on STAT3 transcriptional activities (Figure 4B). IL-6 is the most prominent target of STAT3 and is associated with poor prognosis of lung cancer [19,20]. The mRNA (upper) and secreted protein (bottom) levels of IL-6 were markedly upregulated in A549-shLDOC1-B11 and -D9 relative to those in A549-shCtrl-C9 and -D10 (Figure 4C). The presence of pSTAT3^Y705^, pJAK2, and pSrc was detected in the A549-shLDOC1-B11 and A549-shLDOC1-D9 cells, whereas nearly none of these protein molecules were detected in the A549-shCtrl-C9 and A549-shCtrl-D10 cells (Figure 4D). Treatment with AZD1480 (0.5 μM), the specific inhibitors of JAK2, yielded suppression of pJAK2 without inhibition of pSrc in A549-shLDOC1-D9 (Figure 4E). JAK2 inhibition led to an absence of pSTAT3^Y705^. PP2 (5 μM) treatment considerably reduced pSrc without suppression of pJAK2 and pSTAT3^Y705^ (Figure 5E). These data suggest that the activated JAK2, not Src, was responsible for the phosphorylation of STAT3^Y705^. Treatment with IL-6 (100 ng/mL) increased the expression of pSTAT3^Y705^ and pJAK2. An IL-6-specific neutralizing antibody (250 ng/mL) yielded a drastic reduction in pJAK2 and pSTAT3^Y705^ in the A549-shLDOC1-D9 cells (Figure 4F), indicating that autocrine IL-6 signaling contributes to JAK2/STAT3 activation caused by LDOC1 downregulation. These results indicated the importance of *LDOC1* in regulation of the reciprocal loop of IL-6/JAK2/STAT3.

We evaluated the biological significance of IL-6/JAK2/STAT3 activation in *LDOC1*-mediated malignant progression of the A549 cell line. Treatment with S3I-201(10 μM), an inhibitor of STAT3, reduced the cell cycle progression in A549-shCtrl-D10 and A549-shLDOC1-D9 cells. AZD1480 (0.5 μM) and PP2 (5 μM) only considerably inhibited the cell cycle progression of the A549-shLDOC1-D9 cells, not that of the A549-shCtrl-D10 cells (Figure 4G, left). The neutralizing antibodies considerably inhibited cell cycle progression in the A549-shCtrl-D10 and A549-shLDOC1-D9 cells (Figure 4G, right). Treatment with S3I-201 (10 μM), AZD1480 (0.5 μM), and anti-IL-6 (250 and 500 ng/mL), but not PP2, drastically suppressed the invasion of A549-shLDOC1-D9 cells (Figure 4H). The invasiveness of the A549-shCtrl-D10 cells could be inhibited slightly by AZD1480 (0.5 μM) and anti-IL-6 (250 and 500 ng/mL), but not by S3I-201 or PP2 (Figure 4H). These results suggested that IL-6/JAK2/STAT3 signaling contributes to the cancer progression caused by *LDOC1* deficiency.

### 2.4. LDOC1 Knockdown Augmented Tumorigenesis and Phosphorylated JAK2 and STAT3 in Xenograft Tumor Model of Lung Cancer

We generated single clones of A549-shLDOC1-B11 and -D9, which stably suppressed *LDOC1* expression and their controls, A549-shCtrl-C9 and -D10, from the cell pools of A549-shLDOC1 and A549-shCtrl, respectively. The mRNA and protein levels of *LDOC1* in these clones were confirmed (Figure 5A). After subcutaneous injection, we measured the tumor volume in 10 nude mice, each bearing tumors derived from A549-shCtrl-D10 and A549-shLDOC1-D9 on their left and right back, respectively. Almost all the A549-shLDOC1-D9-derived tumors considerably increased in volume compared with the A549-shCtrl-D10-derived tumors in the same mice, except those in mouse Nos. 7 and 9 (Figure 5B,E). By day 35, the A549-shLDOC1-D9-derived tumors had drastically increased in weight compared with the A549-shCtrl-D10-derived tumors in the same mice, except those in mouse No. 9 (Figure 5C). This notable augmentation in average tumor weight is illustrated in Figure 5D. As shown in Figure 5F, immunohistochemistry results indicated that levels of pJAK2 and pSTAT3^Y705^ were increased in A549-shLDOC1-D9–derived tumors compared with those of A549-shCtrl-C9–derived tumors. This provides evidence for the effect of *LDOC1* on the IL-6/JAK2/STAT3 signaling pathway.

### 2.5. LDOC1 Associated with LNX1 and pJAK2 to Facilitate Ubiquitination–Proteasomal Degradation of pJAK2

**Figure 6:**
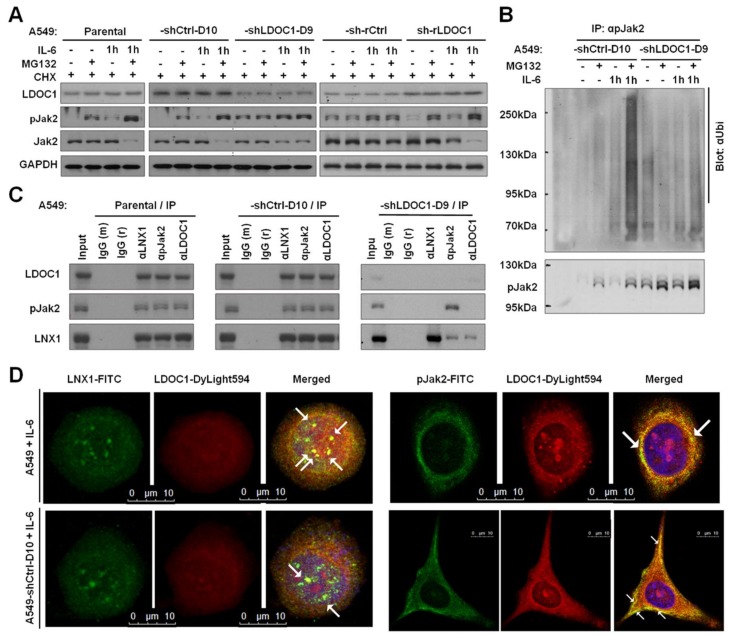
LDOC1 interacts with Ligand Of Numb-Protein X 1 (LNX1) to promote phospho-JAK2 (pJAK2) degradation through the ubiquitin–proteasome pathway. (**A**,**B**) LDOC1 enhances proteasomal degradation (**A**) and ubiquitination (**B**) of pJAK2 in basal and IL-6-stimulated A549-derived cell lines. A549-derived cell lines were starved overnight and pretreated with CHX (100 μg/mL), either alone or with proteasome inhibitor MG132 (20 uM) for 3 h before IL-6 (100 ng/mL) stimulation. The cells were lysed in an NP-40 lysis buffer containing MG132 (20 μg/mL) and ubiquitin aldehyde (20 μg/mL) to inhibit isopeptidase activities. (**A**) Western blotting was performed to examine the levels of indicated proteins. (**B**,**C**) The cell extracts were precleared with mouse or rabbit IgG (IgG^m^ or IgG^r^, respectively) before being immunoprecipitated using antibodies against either pJAK2 (**B**,**C**) or against LDOC1 and LNX1 antibodies (**C**). The input and the Immunoprecipitation (IP) fractions were analyzed through immunoblotting (blot) using antibodies that recognized the indicated proteins. After being stripped, the membrane was reblotted with an anti-pJAK2 antibody (**B**). (**D**) Associations between LDOC1-LNX1 and LDOC1-pJAK2 in IL-6-treated A549 and A549-shCtrl-D10 cells. IL-6 stimulated cells were subjected to immunofluorescence assay (IFA) by using anti-LDOC1-DyLight594 (red), anti-LNX1-FITC (green), and anti-pJAK2-FITC (green), respectively. The 4′,6-diamidino-2-phenylindole (DAPI, blue) was used for nuclear counterstaining. Images were captured using a confocal microscope. IP or IFA performed with IgG^m^ or IgG^r^ were used as negative controls.

We revealed that pJAK2 was responsible for the phosphorylation of STAT3^Y705^ in A549-shLDOC1-D9 (Figure 4E). We examined whether LDOC1 influences the proteasomal degradation of pJAK2. We discovered that in *LDOC1*-highly expressing-A549 and -A549-shCtrl-D10, either intrinsic or IL-6-stimulated pJAK2 efficiently underwent degradation by proteasome and that pJAK2 was nearly undetectable without treatment with proteasome inhibitor MG132 (lanes 1–8 in Figure 6A). By contrast, in *LDOC1*-low expressing-A549-shLDOC1-D9 and -A549-sh-rCtrl cells, abundant pJAK2 remained with or without treatment with MG132 (lanes 9–16 in Figure 6A), indicating that *LDOC1* knockdown led to a notable reduction in the efficiency of pJAK2 proteasomal degradation, whereas restored *LDOC1* expression prevented this inefficiency (A549-sh-rLDOC1, lanes17–20 in Figure 6A). We then examined whether LDOC1 could influence pJAK2 proteasomal degradation by regulating its ubiquitination. As presented in Figure 6B, high levels of ubiquitinated pJAK2 were detected in *LDOC1* expressing-A549-shCtrl-D10 cells but only traces of ubiquitinated pJAK2 were detected in A549-shLDOC1-D9, with or without IL-6 stimulation. These results suggested that LDOC1 promotes ubiquitination of pJAK2. By searching the STRING database (http://string-db.org), we discovered that Ligand Of Numb-Protein X 1 (LNX1), an E3 ubiquitin ligase, is a potential interaction partner of LDOC1. Results from co-immunoprecipitation and Western blotting analysis indicated that pJAK2, LNX1, and LDOC1 formed multiprotein complexes in IL-6-stimulated A549 and A549-shCtrl-D10, whereas only a small fraction of LNX1 co-immunoprecipitated with pJAK2 in the IL-6-treated A549-shLDOC1-D9 cells (Figure 6C), suggesting that LDOC1 deficiency may attenuate the interactions of LNX1-pJAK2. We investigated the LNX1-LDOC1 interactions in the A549 and A549-shCtrl-D10 cells through immunofluorescence confocal microscopy. As shown in Figure 6D, LNX1-LDOC1 and pJAK2-LDOC1 interactions (yellow staining) were observed in the cytoplasm of the A549 and A549-shCtrl-D10 cell lines, which is in agreement with the results of the co-immunoprecipitation experiments. Overall, our data suggested that LDOC1 acts as an adaptor to facilitate LNX1-pJAK2 associations, which promotes pJAK2 degradation and suppresses STAT3 activation in A549 cells.

## 3. Discussion

**Figure 7:**
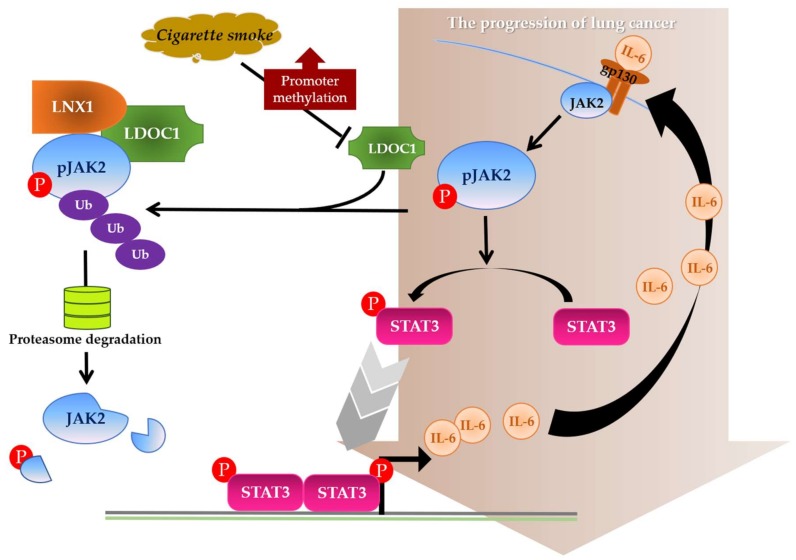
Model outlining the role of *LDOC1* as a cigarette smoke-sensitive negative regulator of IL6/JAK2/STAT3 in lung cancer. LDOC1 functions as an adaptor between pJAK2 and E3 ligase LNX1. After cigarette smoke exposure, *LDOC1* expression was downregulated or silenced by promoter hypermethylation, which led to inefficient ubiquitination of pJAK2 because a LNX1–pJAK2 complex could not form. Accumulated pJAK2 activated STAT3 through phosphorylation of STAT3^Y706^. pSTAT3^Y706^ translocated to the nucleus, where it regulated the expression of target genes such as IL-6. At an increased concentration, IL-6 bound to cell-surface receptor gp130, resulting in the phosphorylation of JAK. In this manner, *LDOC1* knockdown drove lung cancer progression by reinforcing the IL6/JAK2/STAT3 signaling loop.

Persistent activation of STAT3 has been observed in 22–65% of NSCLCs [21,22] and has been proposed as one of the key events in this human malignancy [22,23]. The canonical STAT3 signaling pathway begins with the binding of IL-6 to its cell-surface receptor gp130, leading to tyrosine phosphorylation of the receptor-associated kinases (JAK or Src). The activated kinase, in turn, cross-phosphorylates tyrosine residues onto the cytoplasmic tail of the receptor, followed by recruitment of latent STAT3 protein to the receptor and phosphorylation at STAT3^Y705^ [24]. The pSTAT3^Y705^ homodimerizes and translocates to the nucleus, where it regulates the expression of target genes such as IL-6. The phosphorylation at STAT^S727^ by MAP kinases (ERK, JNK, and p38MAPK) yields a fully activated STAT3 [25]. Activated STAT3 usually has a short lifespan. However, constitutive STAT3 activation can be caused by either the overexpression of gp130 and kinases or attenuated activity of negative regulators, such as suppressor of cytokine signaling proteins, protein inhibitors of activated STAT, and protein phosphatase (e.g., SHP-1 and SHP-2) [26]. Naturally occurring mutations in STAT3 that yield persistently activated STAT3 are rare but can occur [27]. A previous study demonstrated a considerable enrichment of mutations in the JAK2/STAT3 axis in NSCLC tumors; the mutation rate was much higher in tobacco smokers than in never smokers [28]. The JAK2 inhibitor AZD1480 inhibits STAT3 activation in NSCLC and suppresses the growth of xenograft tumors harboring persistent STAT3 activation [29,30,31]. In addition, it was reported that STAT3 is activated by JAK2, independent of other key oncogenic driver mutations in NSCLCs [32]. These findings emphasize the crucial role of JAK2 in STAT3 regulation in tobacco-associated lung carcinogenesis. The present study is the first to illustrate that LDOC1 functions as a bridge between tobacco smoke exposure and IL-6/JAK2/STAT3 activation in lung cancer. We demonstrated that *LDOC1* promoter hypermethylation and downregulation by CSC treatment (Figure 1D) led to the activation of the IL-6/JAK2/STAT3 signaling loop (Figure 4). Although *LDOC1* downregulation was observed in many human malignancies, no genetic mutation of *LDOC1* has thus far been identified in tumors, except in hereditary prostate cancer [33]. This supports our finding that promoter hypermethylation plays a causative role in *LDOC1* silencing in human cancers. In our previous work, the nuclear accumulation of DNA methyltransferase 1 (DNMT1), but not DNMT3a and DNMT3b, substantially increased after both short-term (within 0.5 h) and long-term (14–28 days) treatment of cigarette smoke condensate (CSC) [4]. Simultaneously, *LDOC1* promoter hypermethylation and gene silencing followed the same treatment. Notably, Liu et al. reported that IL-6 increased the expression of DNMT1 but not DNMT3a and DNMT3b and enriched the lung cancer stem-like cell population by inhibiting the cell cycle regulator [34]. These results suggest that DNMT1 may be the main enzyme responsible for *LDOC1* promoter methylation. Moreover, it may further imply that cigarette smoking triggers an oncogenic loop, in which smoking induces DNMT1-mediated promoter methylation and *LDOC1* downregulation, which enhances IL-6/JAK2/STAT3 signaling; subsequently, the increased levels of IL-6 upregulate DNMT1 expression in a feedback mode resulting in *LDOC1* hypermethylation and silencing and the further reinforcement of the IL-6/JAK2/STAT3 axis. Such an oncogenic loop accelerates lung cancer progression (Figure 7). Additional experiments to investigate whether other molecules contribute to the promoter methylation of *LDOC1* are needed. Considering the effect of cigarette smoke-induced epigenetic aberrations on the development and progression of lung cancer, confirming the importance of the methylation-dependent silencing of *LDOC1* in this malignancy by performing in vivo analysis that entails the induction of hypermethylation on the *LDOC1* promoter through CRISPR/Cas9–mediated knockin of DNMTs is necessary. 

The regulation of transcription factors (TFs) can be classified into two principally different levels: intracellular protein concentration and activity, each of which can be modulated in various mechanisms. The activity of a TF is often regulated by phosphorylation or dephosphorylation, which may affect a varied range of functions such as nuclear translocation, DNA binding, and *trans*-activation. Different TFs are subject to different regulatory patterns. As shown in Figure 4A, the DNA-binding activities of several TFs are affected by *LDOC1* suppression. At least 50 LDOC1-interacting partners have been identified thus far, and LDOC1 likely regulates the activity of different TFs through different protein–protein interactions and mechanisms. In the case of STAT3, our data indicate that pJAK2, but not pSrc, is responsible for the phosphorylation of STAT3^Y705^ (Figure 4E) in A549-shLDOC1-D9 cells. *LDOC1* expression has a major effect on pJAK2 stability through the modulation of the ubiquitination of pJAK2 (Figure 6A,B). LDOC1 promotes proteasome degradation of pJAK2 (active form of JAK2) by serving as an adaptor between pJAK2 and E3 ligase LNX1 (Figure 6C,D). Thus, the phosphorylation or transcriptional activity of STAT3 can be switched off, without changing the expression of STAT3 and JAK2, by the actions of LDOC1-mediated proteasome degradation of pJAK2. Similarly, *LDOC1* knockdown has demonstrated a considerable effect on several cellular phenotypes, possibly through the activation of a diverse range of molecules and signaling. Moreover, proliferation, cell-cycle progression, and invasiveness of cancer cells may be regulated by *LDOC1* through distinct signaling pathways. As shown in Figure 4D, both pSrc and pJAK2 were present in A549-shLDOC1 cells but they were nearly absent in A549-shCtrl cells. Activated Src was not responsible for STAT3^Y706^ phosphorylation and did not contribute to the invasiveness of A549-shLDOC1 cells. However, pSrc may play a role in the cell cycle progression of A549-shLDOC1 cells through molecular cascade other than IL-6/JAK2/STAT3. This may explain why PP2 inhibited cell cycle progression (Figure 4G) but had no effect on the invasiveness of A549-shLDOC1 cells (Figure 4H). The findings of Liu et al. [35] support our explanation. That study demonstrated that Src regulates cell cycle protein expression through protein kinase B/glycogen synthase kinase 3 beta and extracellular signal-regulated kinase 1/2 pathways in the human breast cancer cell line MCF-7.

On the basis of the microarray data sets obtained from the study of Bhattacharjee et al. [36], we previously [4] noted that *LDOC1* expression was relatively low in small cell lung carcinoma (SCLC)—a lung cancer type strongly associated with smoking—and non-SCLC (NSCLC). In addition, *LDOC1* was silenced and promoter hypermethylation was present in the NSCLC cell line H1299 (Figure 1B). The ectopic expression of *LDOC1* considerably suppressed the invasiveness of H1299 cells (Figure 3F). Kaplan–Meier analysis demonstrated that patients with stage IV lung cancers, including lung squamous cell carcinoma (LSQC, *n* = 19) and lung adenocarcinoma (*n* = 47), expressing low *LDOC1* levels had a significantly shorter (*p* = 0.004, log-rank test) overall survival than patients expressing high *LDOC1* levels (Figure 2D). These results supported the premise that *LDOC1* is an indicator for tobacco exposure and suggested that *LDOC1* acts as a tumor suppressor in the progression of not only LADC but also NSCLC and LSQC. However, additional experiments are required to confirm the function and mediating mechanism of *LDOC1* in SCLC and LSQC. In addition, both EGFR and ALK play pivotal roles in lung cancer pathogenesis. Interplay has been reported between EGFR, STAT3, and ALK in lung cancers [37,38,39,40]. Therefore, STAT3 activation induced by *LDOC1* silencing may be associated with genetic abnormalities of EGFR and ALK and may exert a synergistic effect in lung cancer; this topic may be worth exploring in the future. Notably, although the IL-6/JAK2/STAT3 axis is generally recognized as a tumor-promoting signaling loop in the progression of lung cancer, its effect in the initiation of lung carcinogenesis remains controversial because IL-6 prevents the initiation of lung carcinogenesis, as evidenced by Qu et al. [41]. Because *LDOC1* is a regulator of the IL-6/JAK2/STAT3 axis, *LDOC1* may play different roles during different stages of lung cancer.

The importance of phosphorylated STAT3^S727^ (pSTAT3^S727^) has been observed; pSTAT3^S727^ is highly enriched in mitochondria than that present in the cytoplasm [42,43], and the activities of mitochondrial STAT3 (mitoSTAT3) are supported by phosphorylation on STAT3^S727^ [43]. The discovery of mitoSTAT3 opened up a new avenue through which STAT3 may regulate cell metabolism and mitochondrial gene expression [42,44]. Within the mitochondria, STAT3 modulates two major players in this cellular compartment: the electron transport chain (ETC) and mitochondria permeability transition pore [42,45]. MitoSTAT3’s influence on altered glycolytic and oxidative phosphorylation activities cause it to play a significant role in tumorigenesis. Our preliminarily data indicated that *LDOC1* knockdown yielded a drop in reactive oxygen species (ROS) in lung cancer cells. The reduction of ROS may be a consequence of enhanced ETC activities. Whether *LDOC1* also regulates the phosphorylation of STAT3^S727^ as well as STAT3^Y705^ warrants further investigation, as does whether this has a profound influence on lung tumorigenesis.

In summary, we demonstrated that promoter hypermethylation and silencing of *LDOC1* in human bronchial epithelial cells followed CSC exposure. LDOC1 interacts with pJAK2 and LNX1 to facilitate ubiquitination and proteasome degradation of pJAK2, which suppress IL-6/JAK2/STAT3 signaling and alleviate the aggressiveness of LADC cell line A549 (Figure 7). This study highlighted that *LDOC1* acts as a novel native negative regulator of JAK2 and STAT3 in lung cancer. Furthermore, this study expands the knowledge of how cigarette smoke promotes lung tumorigenesis. In particular, the data revealed a novel regulatory mechanism of the IL-6/JAK2/STAT3 loop in the pathogenesis of lung cancer.

## 4. Methods

### 4.1. Chemicals and Reagents

5-Aza-2-deoxycytidine (5-AzaC) and cycloheximide (CHX) were purchased from Sigma-Aldrich (St Louis, MO, USA). MG132 was purchased from ApexBio. Inhibitors of STAT3 (S3I-201), JAK2 (AZD1480), and Src (PP2) were purchased from Selleckchem (Selleckchem Chemicals, Houston, TX, USA). The cytotoxicity of S3I-201, AZD1480, and PP2 was measured by the MTT method (Figure S1). Antibodies against STAT3 (79D7), pSTAT3^Y705^ (D3A7), JAK2 (D2E12), pJAK2 (3771), Src (36D10), and α-tubulin (2144) were purchased from Cell Signaling; anti-LNX1 (NBP1-49975, Novus, Littleton, CO, USA); anti-pSrc (9A6, Millipore, Billerica, MA, USA); anti-pJAK2 (PJAK2-240AP, FabGennix, Frisco, TX, USA); anti-GAPDH (GTX100118, GeneTex, Hsinchu, Taiwan); anti-LDOC1 (2507C1a, Santa Cruz, Dallas, TX, USA) for immunoprecipitation and immunofluorescent assay (IFA); anti-LDOC1 (LS-B3527, LifeSpan, Providence, RI, USA) for immunohistochemistry (IHC) study, and anti-β-actin (AC-15, Novus).

### 4.2. Preparation and Treatment of Cigarette Smoke Condensate (CSC)

Preparation of CSC was performed as previously described [4]. The CSCs were dissolved in DMSO. BEAS-2B cells were constantly treated with 0.01, 0.1, or 1 μg/mL of CSC; and fresh medium containing CSC was replaced every 3 days. The DMSO-treated cells were used as controls. At appropriate times, cells were harvested and processed for further analysis.

### 4.3. Cell Lines and Transfection

Authentication of BEAS-2B and A549 cell lines were provided in April 2018 by Topgen Biotechnology Co., Ltd. (Kaohsiung City, Taiwan) using DNA profiling-short tandem repeats (STR) method (Supplementary Materials). The human bronchial epithelial cell line BEAS-2B was purchased from Food Industry Research and Development Institute (Hsinchu City, Taiwan) and was maintained in LHC-9 medium (GIBCO, Waltham, MA USA). The lung adenocarcinoma (LADC) cell lineA549 (originally purchased from ATCC) was maintained in Dulbecco’s modified Eagle medium (DMEM, Sigma-Aldrich, St. Louis, MO, USA) supplemented with 10% FBS, penicillin (100 U/mL), and streptomycin (100 μg/mL). NSCLCs cell lines H460, H1299, and H1355 were maintained in RPMI1640 (Sigma-Aldrich) medium with the same additives. Cells were maintained at 37 °C in a humidified 5% CO_2_ incubator. For knockdown or re-stored expression experiments, A549 cells were transfected with lentiviral pGIPZ carrying either shRNA vectors targeting human LDOC1 (shLDOC1) or GFP-tagged LDOC1 ORF (GE Dharmacon, Chicago, IL, USA), respectively. The cell pools of A549-shLDOC1 and A549-sh-rLDOC1 were selected with 5 µg/mL puromycin (Sigma-Aldrich) and 20 μg/mL blasticidin S (Invitrogen, Waltham, MA, USA), respectively.

### 4.4. Bisulfite Conversion of Genomic DNA and Quantitative Methylation-Specific PCR (qMSP)

The bisulfite conversion and pMSP experiments were performed using a BisulFlash DNA modification kit (Epigentek, Farmingdale, NY, USA) and SYBR Advantage qPCR Premix (Clontech, Mountain View, CA, USA), respectively, as described previously [3]. The primer sequences were shown in Table S1.

### 4.5. Quantitative Real-Time PCR (qPCR)

Total RNA was isolated using RNeasy Mini Kits (Qiagen, Hilden, Germany) according to the manufacturer’s instruction and 18S rRNA expression was used as an internal control. The primer sequences for LDOC1, IL-6, and 18S rRNA are listed in Table S2.

### 4.6. Study Subjects and Human Tissue Microarray (TMA) Constructs

A total of 134 specimens from human primary NSCLC tumors, comprising 82 adenocarcinoma (AD) and 52 squamous cell carcinoma (SQ) specimens, that were histopathologically identified at Chung Shan Medical University Hospital between 1998 and 2008, were used in this study. The surgical samples for the primary NSCLC were freshly collected by routine tissue processing including formalin fixation and paraffin embedment. Tissue cores punched from paraffin blocks, with a diameter of 1.5 mm or 2 mm, were used to construct the TMAs. This study was approved by the Institutional Review Board of the China Medical University Hospital (number: CMUH103-REC2-067). Informed consent was obtained from each patient.

### 4.7. Immunohistochemistry (IHC) and Quantitative Staining Measurement of IHC

The TMA sections (4 μm) were routinely processed and stained using anti-LDOC1 (Acris Antibodies), biotinylated immunoglobulins, and a super-sensitive HRP label system as previously described [3]. The immunoreactivity for LDOC1 was assessed by using the software-based image analysis H-scoring system (Aperio Technologies, Vista, CA, USA). The grades of LDOC1 staining intensity were four-tiered as follows: 0, negative staining; 1, weak staining; 2, medium staining; and 3, strong staining. The H-score of each tissue core represented the sum of the mean value multiplied by the proportion of positive cells in each grade of staining intensity. The H-scores ranged from 0 to 300. The optimal cut-off score determined by ROC analysis was 210 to discriminate high and low levels of LDOC1 expression.

### 4.8. Cell Viability and Proliferation Assay

Cell viability and proliferation were measured as previously described [46].

### 4.9. BrdU Incorporation Assay

BrdU incorporation assay was performed as previously described [46].

### 4.10. Trans-Well Invasion Assay

Experiments were performed as previously described [47]. Briefly, cells were incubated with or without the indicated concentrations of the inhibitor or anti-IL-6 antibodies for 4 h before seeding onto the Matrigel-coated chamber (BD Biosciences, Franklin Lakes, NJ, USA). After 16 h, the invading cells were stained with crystal violet and counted. Data were averaged for three independent experiments, each with four replicates.

### 4.11. Soft Agar Assay

Experiments were performed and quantified as previously described [47].

### 4.12. Transcription Factor (TF) Profiling Array

Cellular nuclear proteins were harvested using a Nuclear Extraction Kit (Chemicon, Rolling Meadows, IL, USA). Experiments were carried out with The TF Activation Profiling Plate Array system (Signosis, Santa Clara, CA, USA) following the manufacturer’s instructions. The DNA binding activities of TFIID was used for normalization. 

### 4.13. Luciferase Reporter Assay

Cells expressing luciferase under STAT3 promoter were generated by transducing lentivirus carrying STAT3-luciferase constructs (Cignal lenti reporter system; Qiagen), following the manufactures’ instruction. 

### 4.14. IL-6 ELISA Assay

The concentration of secreted IL-6 was measured using a human IL-6 ELISA kit according to the manufacturer’s instruction (R&D Systems, Minneapolis, MN, USA).

### 4.15. Immunoprecipitation and Western Blot Analysis

Experiments were carried out as previously described [48]. For anti-ubiquitin detection, immunoprecipitation were performed in the presence of 25 μM MG132 and Western blotting was carried out as described previously [48]. The membrane was incubation with stripping buffer (Thermo Fisher Scientific, Waltham, MA, USA) and for re-probing with antibodies as indicated.

### 4.16. Mouse Xenogaft Tumor Model

All animal experiments were conducted under a protocol approved by the Laboratory Animal Center of National Health Research Institutes. Mice were housed in a temperature and humidity controlled room maintained on standard rodent chow with unrestricted access to water. A549-shCtrl-D10 and A549-shLDOC1-D9 lung cancer cells (5 × 10^6^ per mouse), respectively, were mixed with equal volumes of Matrigel (#356237, Corning, Corning, NY, USA) and injected *subcutaneous* (s.c.) into the left and right dorsal flank area, respectively, of female BALB/C (nu/nu) nude mice (*n* = 10) aged 8 weeks. Tumors were measured by a caliper and the volume was calculated using *V* = A × B^2^ × 0.5 (A, long diameter; B, short diameter). Tumors were harvested from euthanized mice 35 days post injection, snap-frozen in liquid nitrogen, and stored in –80 °C. For IHC studies, formalin fixation followed by paraffin embedment was performed.

### 4.17. Immunofluorescence Assay (IFA) and Confocal Microscopy

Cells were seeded at 20–30% confluence 16 h before IFA. Cells were fixed and permeabilized by exposure to 100% methanol for 12 min at room temperature. After a rehydration with multiple rinses in PBS, the cells were incubated with PBS containing 1% bovine serum albumin to block nonspecific binding. Antibodies were diluted at 1:50 and incubated for 16 h at 4 °C. After three washes with PBS, cells were probed with fluorescent (DyLight 594 or FITC)-conjugated secondary antibodies. Nuclei were counterstained with DAPI (Invitrogen). The samples were analyzed under a laser scanning confocal microscope (Leica TCS SP5, Wetzlar, Germany).

### 4.18. Statistical Analysis

Comparisons of the results between various experimentally treated groups and their corresponding controls were carried out by Student’s *t*-test. Data presented are the means± SD of *n* independent experiments as described in the Figure legends. Statistical significance was considered difference when *p* < 0.05 and significance when *p* < 0.01.

### 4.19. Availability of Data and Materials

An online Kaplan–Meier overall survival (OS) analysis of an integrated database of ten published transcriptomic datasets of lung cancer [18] (http://kmplot.com/analysis).

## 5. Conclusions

In this study, we demonstrated the tumor suppressor function of LDOC1 in lung cancer. These data unveil the molecular mechanism through which LDOC1 inhibits the reciprocal loop of IL-6/JAK2/STAT3. Therefore, epigenetically silencing of LDOC1 by tobacco exposure promotes lung cancer progression and suggests that LDOC1 downregulation is a biomarker associated with poor survival of patients with lung cancer.

## Supplementary Materials

The following are available online at http://www.mdpi.com/2072-6694/11/1/63/s1, Figure S1: The cytotoxicity of S3I201, AZD1480, and PP2 on lung cancer cell lines A549-shCtrl-D10 and A549-shLDOC1-D9. Table S1, Information on qMSP primers for LDOC1; Table S2, qPCR primers for LDOC1 and IL-6.
