# Supplementary material for: Novel STAT3 Inhibitor LDOC1 Targets Phospho-JAK2 for Degradation by Interacting with LNX1 and Regulates the Aggressiveness of Lung Cancer
Source: Cancers (Basel). 2019 Jan 9;11(1):63. doi: 10.3390/cancers11010063 (PMC6356782; doi:10.3390/cancers11010063)
Supplement: Supplementary file 1 [file cancers-11-00063-s001.pdf]

# Supplementary Materials: Novel STAT3 Inhibitor LDOC1 Targets Phospho-JAK2 for Degradation by Interacting with LNX1 and Regulates the Aggressiveness of Lung Cancer

**Chia-Huei Lee, Ji-Rui Yang, Chih-Yu Chen, Ming-Hsien Tsai, Pin-Feng Hung, Shin-Jih Chen, Shang-Lun Chiang, Han Chang and Pinpin Lin**

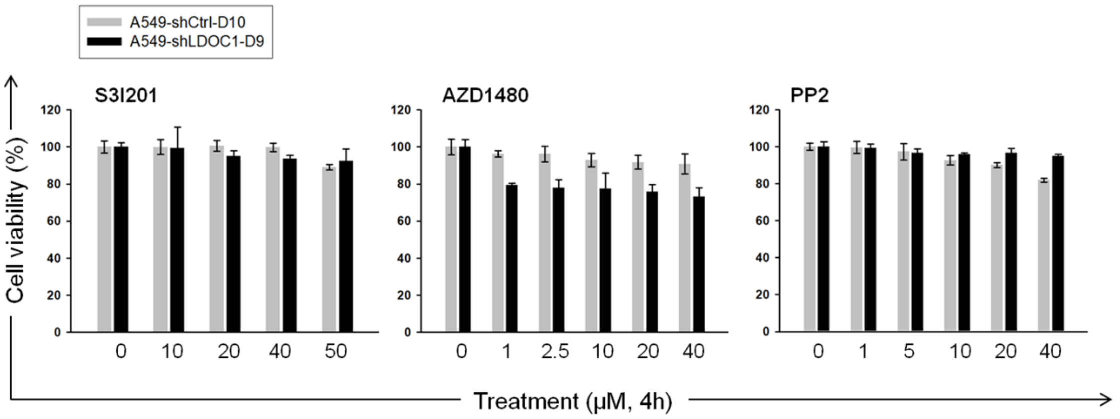


**Figure S1.** The cytotoxicity of S3I201, AZD1480, and PP2 on lung cancer cell lines A549-shCtrl-D10 and A549-shLDOC1-D9. Cell viability was assessed using an MTT assay after a 4-h pre-treatment with the indicated concentrations of inhibitors of STAT3 (S3I201), Jak2 (AZD1480), and Src (PP2).

**Table S1.** Information on qMSP primers for LDOC1.

| Regions | Primers | Sequence 5'-3' | Location [a] | Amplicom (bp) | # CpG [b] |
|---|---|---|---|---|---|
| mLDOC1-1 | mLDOC1-4F | 5′-CGGTGTTTTTTTTCGAAAC-3' | −460 | 108 | 14 |
| | mLDOC1-4R | 5'-CGTCGTTACAAAATCGATTC-3' | −567 | | |
| mLDOC1-2 | mLDOC1-3F | 5'-CGAGAGGGTTAGTTTGTTGC-3' | −417 | 126 | 15 |
| | mLDOC1-3R | 5'-ACGAACATATAAAACGCCGT-3' | −542 | | |
| mLDOC1-3 | mLDOC1-2F | 5'-CGTGGATACGTATTTTTTGC-3' | −227 | 128 | 13 |
| | mLDOC1-2R | 5'-CATCAAAAACGCGTACAAC-3' | −354 | | |
| mLDOC1-4 | mLDOC1-1F | 5'-TGAGATCGGATTTAGCGTTC-3' | −6 | 130 | 8 |
| | mLDOC1-1R | 5'-CTAAATCGCTAAACCACCGA-3' | 124 | | |

[a] The 5' nucleotide number where the primer begins (from transcription start site). [b] Amount of CpG dinucleotides.

**Table S2.** qPCR primers for LDOC1, IL-6, and 18S rRNA.

| Symbol | Primers | Sequence 5'-3' |
|---|---|---|
| LDOC1 | LDOC1-F | 5'-ATGACGACGAAGACGACGA-3' |
| | LDOC1-R | 5'-GAGGGTCGAGGGCCTAATAA-3' |
| IL-6 | IL-6-F | 5'-GATGAGTACAAAAGTCCTGATCCA-3' |
| | IL-6-R | 5'-CTGCAGCCACTGGTTCTGT-3' |
| 18S rRNA | 18Sr-F | 5'-TGTGCCGCTAGAGGTGAAATT-3' |
| | 18Sr-R | 5'-TGGCAAATGCTTTCGCTTT-3' |

© 2019 by the authors. Submitted for possible open access publication under the terms and conditions of the Creative Commons Attribution (CC BY) license (http://creativecommons.org/licenses/by/4.0/).
